# Sjogren’s Syndrome: Recent Updates

**DOI:** 10.3390/jcm11020399

**Published:** 2022-01-13

**Authors:** Charalampos Skarlis, Sylvia Raftopoulou, Clio P. Mavragani

**Affiliations:** 1 Department of Physiology, School of Medicine, National and Kapodistrian University of Athens, 11527 Athens, Greece; charskarlis@med.uoa.gr (C.S.); sylviaraft98@gmail.com (S.R.); 2 Fourth Department of Internal Medicine, School of Medicine, University Hospital Attikon, National and Kapodistrian University of Athens, Haidari, 12462 Athens, Greece; 3 Joint Academic Rheumatology Program, School of Medicine, National and Kapodistrian University of Athens, 11527 Athens, Greece

Primary Sjögren’s syndrome (SS) is a chronic systemic autoimmune disorder affecting primarily perimenopausal women. The key histopathological features of SS are mouth and eye dryness as a result of periductal lymphocytic cell infiltration in salivary and lacrimal glands [1,2]. B cell hyperactivity, serum autoantibody over secretion (anti-Ro/SSA, anti-La/SSB), rheumatoid factor (RF), as well as activation of type I and II interferon (IFN) pathways are established disease hallmarks [3,4,5,6]. The most severe complication of SS is non-Hodgkin’s Lymphoma (NHL) development (with an estimated relative risk of 4 to 40-fold higher than that of the general population); it occurs in 5–10% of primary SS patients, representing the leading cause of SS-related mortality [7,8,9].

Despite the progress in understanding SS immunopathological mechanisms, the role of innate immunity in disease development remains unelucidated. In this context, type I IFNs have attracted a great research interest. IFNs are functionally related cytokines of innate immunity, exerting antiviral, antimicrobial, antitumor and immunomodulatory activities. Cumulative evidence revealed dysregulation of type I IFNs as a major pathogenetic mechanism in the development of several autoimmune diseases including SS. The majority of patients exhibit persistent systemic activation of the type I interferon (IFN) system, a feature shared with other systemic autoimmune diseases [6]. In their comprehensive review, Huijser and Versnel highlight the role of deregulated intracellular sensing of viral or endogenous nucleic acids by RNA and DNA intracellular receptors in the sustained production of type I IFN in the setting of SS [10]. Moreover, Sebastian and colleagues report that SS patients positive for IFNγ (cut off = 36.57 pg/mL serum levels) are younger (˂43 years) and display higher RF titers and EULAR Sjögren’s syndrome disease activity index (ESSDAI) compared to IFNγ negative subjects [11].

Increased apoptosis of salivary gland epithelial cells has been postulated as a key pathogenetic event in the setting of SS with increased exposure of endogenous autoantigens leading to generation of a localized immune response. Insulin-like growth factor (IGF) and its receptor (IGF1R) contribute to cellular homeostasis by regulating apoptosis. As IGF1R mRNA and protein expression levels were found to be decreased in salivary gland tissues from SS patients in negative association with caspase 1 expression levels, we hypothesized that defective IGF1 mediated trophic signals in salivary glands can lead to pyroptosis through activation of the NLRP3-inflammasome pathway. Notably, genetic variation of the IGF1R rs2229765 variant was found to be associated with increased susceptibility for seropositive primary SS [12].

Oral dryness resulting from reduced saliva production due to focal lymphocytic infiltration of salivary and lacrimal glands, is one of the key SS clinical manifestations. In an effort to elucidate the underlying mechanisms responsible for hyposalivation in SS, Blochowiak and colleagues employed high throughput microarray assays to demonstrate that genes related to endoplasmic reticulum (ER) function could be involved in SS-related xerostomia in labial salivary gland tissues derived from SS patients with sicca symptoms compared to their counterparts with no sicca complaints as well as healthy controls [13]. In line with previous observations highlighting the role of endoplasmic stress in SS pathogenesis [14], the authors suggest that decreased expression of such genes may result in the accumulation of misfolded proteins in the ER and therefore in the alteration of quality and quantity of salivary proteins leading to SS-related xerostomia.

Focal lymphocytic sialadenitis (FLS) the SS histopathological hallmark is characterized by periductal focal lymphocytic salivary gland infiltrates. It was previously shown in [15] that minor salivary gland (MSG) infiltrates mainly consist of T (predominantly CD4^+^) and B lymphocytes with the ratio of T/B cells having a negative association with infiltration grade and biopsy focus score. Joachims and colleagues evaluated salivary gland (SG) biopsies for the distribution of infiltrating CD4^+^ and CD8^+^ T cells by utilizing flow cytometry and microarray analysis. In line with previous findings, it has been shown that CD4^+^ memory T cells infiltrates are increased in salivary gland tissues of primary SS patients in association with distinct clinical and serological features such as corneal damage and serum antibody levels. They also exhibit an enrichment pattern for a germinal center Tfh profile, compared to non-SS sicca controls [16].

From an orthopedic viewpoint, systemic SS manifestations involve the musculoskeletal system [17,18] affecting bones, muscles and specific joints. In the current Special Issue, Rozis et al. have summarized the current knowledge on SS-related musculoskeletal manifestations including arthritis, muscle and peripheral nervous system involvement as well as bone metabolism alterations [19]. 

Among other comorbidities and similarly to other autoimmune diseases such as systemic lupus erythematosus (SLE) and rheumatoid arthritis (RA), SS is associated with increased risk for atherosclerosis and coronary artery disease (CAD) [20,21,22,23]. In SLE, type I IFN has been shown to be associated with endothelial dysfunction—a pathogenetic contributor of atherosclerosis. On this basis, Yang and colleagues conducted a retrospective cohort study in a Taiwanese population to examine whether treatment with hydroxychloroquine (HCQ), an antimalarial agent used as immunomodulatory treatment in autoimmune diseases, can confer protection against CAD in SS patients using HCQ long-term [24]. They concluded that the incidence of CAD was significantly lowered in SS patients that underwent long-term HCQ treatment.

In the setting of SS, optic neuropathy is a rare complication; however, it might occur after chronic HCQ treatment [25]. Dziadkowiak and collaborators explored the diagnostic usefulness of visual evoked potentials (VEP) examination, in patients with primary Sjögren’s Syndrome (SS), without focal symptoms suggesting the presence of underlying central nervous system disorder. Of interest, their data showed an association between abnormalities in electrophysiological parameters of VEP at baseline with the presence of anti-Ro52 antibodies and aching joints.

Among the more serious disease complications of SS is non-Hodgkin’s lymphoma (NHL) and, more frequently, mucosa-associated lymphoid tissue (MALT) lymphoma compared to all other autoimmune disorders, leading to increased morbidity and mortality rates. Identification of valuable predictive markers for lymphoma development and prognosis remains challenging. This Special Issue includes a literature review by Stergiou et al. which summarizes the main pathogenetic mechanisms involved in the SS-related lymphomagenesis [26]. Briefly, the process of lymphomagenesis may be initiated when lymphoepithelial sialadenitis is coupled by sustained antigenic stimulation leading to emergence of autoreactive B cell clones in salivary glands of SS patients. Formation of ectopic germinal centers along with several oncogenic events are potential drivers of malignant transformation.

Although SS prevalence in males is lower compared to females, male gender has been associated with higher susceptibility for lymphoma development compared to females. To investigate further the role of epidemiological factors in SS related lymphoma, Chatzis and colleagues conducted a multicenter prospective cohort study, restricted in SS male patients in order to identify possible biomarkers for lymphoma development in the male patient subgroup [27], confirming that male SS patients were at higher risk for developing SS-related lymphoma compared to females.

The heterogeneous nature of the clinical SS features, as well as NHL development, generated the need to focus on advanced techniques for predicting the risk for lymphoma development in primary SS patients. Salivary gland enlargement is an adequate predictive marker for possible lymphoma development, therefore imaging and biopsy techniques represent a valuable tool for the SS-related lymphoma prediction [28,29]. Manfrè et al. summarized the role of different imaging techniques and a bioptic approach in pSS patients, focusing mainly on the role of salivary gland ultrasonography (SGUS) and a US-guided core needle biopsy as diagnostic and prognostic tools in primary SS patients with persistent parotid swelling [30].

Over the last years, an increasing number of studies revealed key roles of numerous clinical, epidemiological, molecular and genetic contributors in SS pathogenesis and disease related lymphomagenesis [9,31]. Of interest, our research group explored the role of the Leukocyte immunoglobulin-like receptor A3 (LILRA3) gene variant—previously shown to confer increased susceptibility for both SS and non-Hodgkin B-cell lymphoma (B-NHL) in the general population- in SS-related lymphomagenesis. Our findings suggest that the functional LILRA3 gene variant increases susceptibility to SS-related lymphoma development in patients with young disease onset of <40 years old, implying that genetically determined deranged immune responses in younger SS individuals could underly their pronounced risk for lymphoma development [32].

Given the wide spectrum of clinical manifestations of SS, disease diagnosis can often be challenging. Despite the incomplete understanding of underlying pathogenetic mechanisms, great progress has been made to understand pathogenesis, clinical spectrum, diagnosis and therapeutic interventions in Sjogren’s syndrome.
